# Cortical and white matter anatomy relevant for the lateral and superior approaches to resect intraaxial lesions within the frontal lobe

**DOI:** 10.1038/s41598-022-25375-z

**Published:** 2022-12-10

**Authors:** Tomasz Andrzej Dziedzic, Aleksandra Bala, Artur Balasa, Agnieszka Olejnik, Andrzej Marchel

**Affiliations:** 1 Department of Neurosurgery, Medical University of Warsaw, Banacha 1a, 02-097 Warszawa, Poland; 2 Faculty of Psychology, University of Warsaw, Warsaw, Poland

**Keywords:** Surgical oncology, Brain

## Abstract

Despite being associated with high-order neurocognitive functions, the frontal lobe plays an important role in core neurological functions, such as motor and language functions. The aim of this study was to present a neurosurgical perspective of the cortical and subcortical anatomy of the frontal lobe in terms of surgical treatment of intraaxial frontal lobe lesions. We also discuss the results of direct brain mapping when awake craniotomy is performed. Ten adult cerebral hemispheres were prepared for white matter dissection according to the Klingler technique. Intraaxial frontal lobe lesions are approached with a superior or lateral trajectory during awake conditions. The highly eloquent cortex within the frontal lobe is identified within the inferior frontal gyrus (IFG) and precentral gyrus. The trajectory of the approach is mainly related to the position of the lesion in relation to the arcuate fascicle/superior longitudinal fascicle complex and ventricular system. Knowledge of the cortical and subcortical anatomy and its function within the frontal lobe is essential for preoperative planning and predicting the risk of immediate and long-term postoperative deficits. This allows surgeons to properly set the extent of the resection and type of approach during preoperative planning.

## Introduction

**Figure 1:**
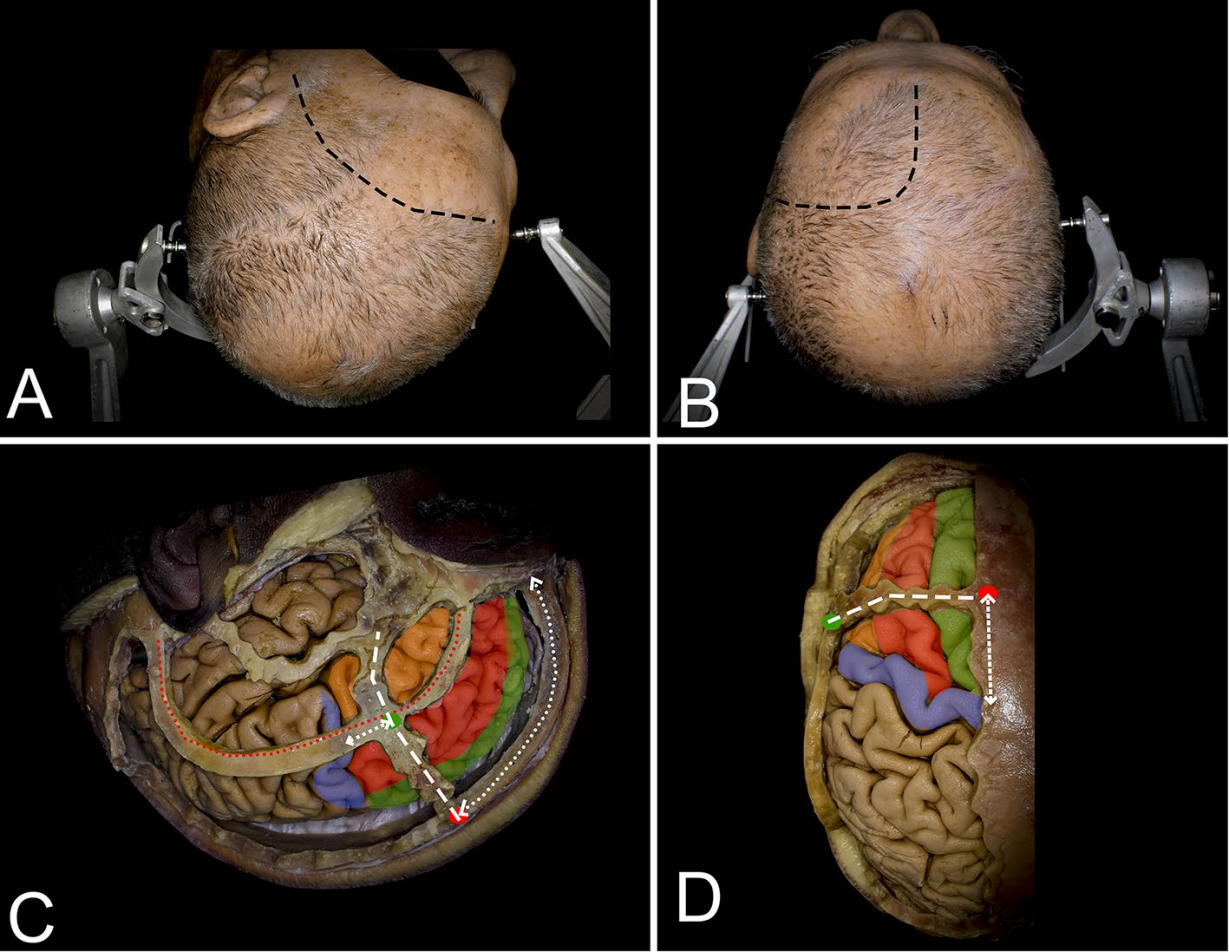
Figure presenting the surgical perspective of the lateral (**A,C**) and superior (**B,D**) approaches to the frontal lobe. (**A**) For the lateral approach, the head is rotated contralateral to the operated side, and a curved skin incision (black dashed line) is made in the frontotemporal region within the hairline, allowing proper exposure of the basal regions of the frontal lobe. (**B**) For the superior approach, the head is positioned in the neutral position, and a curved skin incision (black dashed line) within the hairline is made. c, d. Most of the frontal lobe is identified anterior to the coronal suture (white dashed line). The posterior part of the frontal lobe, including the precentral gyrus (blue), is located behind the coronal suture, and on the midline, the precentral gyrus is located approximately 4.5 cm behind bregma (red dot), while on the lateral surface, the precentral gyrus is located in proximity to the lateral sulcus 2.5 cm behind the stephanion (green dot). The coronal sutures are usually easily palpable through the skin, but in difficult cases, they can be identified approximately 13 cm behind the nasion along the midline (white dotted line)^13,27,35^. The inferior frontal gyrus (orange) is identified below the superior temporal line (red dotted line). The pars opercularis of the IFG is located behind the coronal suture, while the pars triangularis and orbitalis are located anteriorly. The middle (red) and superior frontal gyri (green) are located between the midline and the superior temporal line.

**Figure 2:**
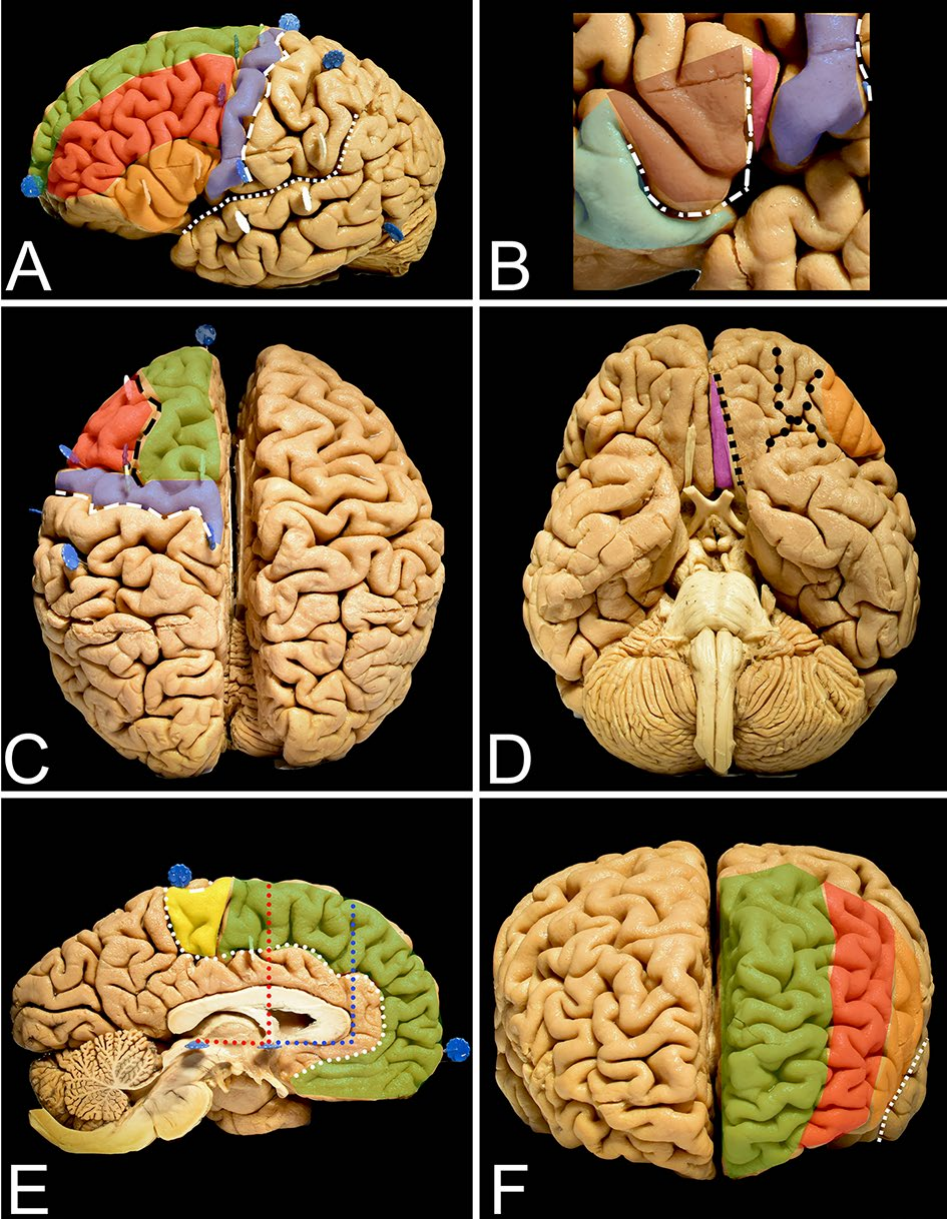
Figure describes. (**A**) The lateral surface is localized anterior to the central sulcus (white dashed line), below the superior margin of the hemisphere and superior to the lateral sulcus (white square-dotted line) and free margin. The vertically oriented precentral gyrus (blue) containing the primary motor cortex is anterior to the central sulcus. The three horizontally oriented gyri of the frontal lobe (superior—green, middle—red and inferior—orange) separated by the superior and inferior sulci are located anterior to the precentral sulcus. (**B**) The inferior frontal gyrus is limited by the lateral sulcus inferiorly and inferior frontal sulcus superiorly and is divided into the pars orbitalis (light blue), pars triangularis (burgundy) and pars opercularis (pink) by the horizontal (white two-dash line) and ascending rami (white dot-dash line) of the lateral sulcus. (**C**) From the superior perspective, mainly the superior and middle gyri, which are separated by the superior frontal sulcus (black dashed line). (**D**) The basal surface of the frontal lobe is formed by a thin medially located gyrus rectus (purple), and a larger lateral part is formed by the four orbital gyri which are separated by the H-shaped orbital sulcus (black dotted line). The border between the lateral and medial parts is marked by the olfactory sulcus with the olfactory tract (black square dot line). (**E**) The mesial surface of the frontal lobe is formed by the paracentral lobule (yellow), superior frontal gyrus and cingulate gyrus, which are separated by the cingulate sulcus (white dotted line). The SMA proper is defined as the region anterior to the motor cortex. The anterior border is set by an imaginary line (red dotted line) perpendicular to the line connecting the anterior and posterior commissure, at the level of the anterior commissure. The anterior border of the pre-SMA is less defined, and it is marked by a line (blue dotted line) parallel to the previous and tangential to the most rostral part of the corpus callosum. f. The anterior surface of the lateral portion of the hemisphere is formed by the superior, middle and inferior frontal gyri, and the lateral sulcus is located laterally.

The frontal lobe is the largest of all five lobes of the human brain, and despite high-order neurocognitive functions such as attention, executive cognition and social behavior, this lobe plays an important role in core neurological functions such as motor control and language^1^. The cortical regions responsible for these primary functions are located within the precentral gyrus (motor area), premotor area and pars opercularis of the inferior frontal gyrus (IFG) (Figs. 1 and 2). Primary low-grade gliomas (LGGs) in the frontal lobe are predicted to involve the supplementary motor area (SMA) complex, while primary insular gliomas may extend into the opercular or frontopolar parts of the frontal lobe^2–4^. The aim of surgical treatment for LGGs, despite descriptions in representative material for neuropathological studies, is to perform maximal safe resection while sparing cortical and subcortical areas and avoiding permanent motor, language, and executive function deficits^5,6^. The extent of resection is important in terms of neurooncological outcome and has a direct impact on overall and progression-free survival as well as on quality of life^7–9^. The risk of surgical treatment for gliomas within the SMA complex is related mainly to loss of the psychomotor drive as well as motor and language deficits, which are often temporary; such injury to the operculum in the dominant hemisphere mainly leads to permanent language disturbances, and injuries within the prefrontal cortex lead to executive dysfunctions^10–12^. There is ongoing discussion about the indications for and benefits of procedures performed within the frontal lobe during awake conditions with intraoperative brain mapping. There is less doubt for gliomas involving opercular regions (Case 1, Fig. 6) of the frontal lobe in the dominant hemisphere, where language deficits can be permanent, than for gliomas in proximity to the SMA complex (Case 2, Fig. 7), as the related deficits are often temporary^11^. Preoperative functional and diffusion tensor tractography neuroimaging as well as intraoperative neuronavigational systems are helpful, but understanding the anatomical background is still mandatory for planning and intraoperative decision-making during direct brain stimulation. The aim of the study was to review the cortical and subcortical anatomy of the frontal lobe, to discuss a strategy for intraoperative mapping and to perform morphometric analysis related to resection performed from lateral and superior trajectories.

## Results

Measurements obtained during the study are summarized in Table 1 for the superior approach and in Table 2 for the lateral approach.

**Table 1:** Measurements related to the superior approach.

	Average	Range
**Superior approach**		
*Lateral surface*		
The central sulcus from the frontal lobe base	137.9 mm	121.1–156.4 mm
The central sulcus to the IHF	69.4°	56°–83°
The precentral sulcus to the central sulcus at the SRP	20.8 mm	14.4–31.1 mm
The precentral sulcus to the central sulcus at the IRP	10.6 mm	4.8–15.4 mm
Length of the paracentral lobule	34.9 mm	20.6–40.7 mm
Length of the superior frontal gyrus corresponding to SMA	17.9 mm	7.5–24.1 mm
Length of the superior frontal gyrus corresponding to preSMA	33.7 mm	29.4–39.5 mm
Superior frontal point to the IHF	26.7 mm	18.4–32.3 mm
Inferior frontal point to the IHF	50.0 mm	41.0–55.2 mm
*Frontal lobectomy*		
**Resection tangential to the genu of the corpus callosum**		
Angle	53.2°	40°–65°
Length	87.6 mm	84–92 mm
**Resection perpendicular to the corpus callosum**		
Resection along the corpus callosum	34.6 mm	28–46 mm
From the genu to the skull base	33.4 mm	30–36 mm
**Resection on the lateral surface**		
Aiming anterior to the pars opercularis	72°	60°–85°
Aiming anterior to the pars triangularis	54°	45°–75°

**Table 2:** Measurements related to the lateral approach.

	Average	Range
**Lateral approach**		
*Lateral surface*		
Length of the horizontal rami of the lateral sulcus	15.2 mm	10.9–21.0 mm
Length of the ascending rami of the lateral sulcus	18.9 mm	9.5–21.9 mm
Length of the pars triangularis	19.8 mm	4.8–31.8 mm
Length of the pars opercularis	7.5 mm	4.9–14.4 mm
Depth to the UF/IFOF complex from cortical surface	21.8 mm	17–26 mm
Depth to the midline from the UF/IFOF complex	23.2 mm	19–27 mm
*Frontal lobectomy*		
**Resection anterior to the pars triangularis**		
Angle	68°	60°–80°
Length	87.6 mm	78–95 mm
**Resection anterior to the pars opercularis**		
Angle	80.4°	75°–85°
Length	82.0 mm	71–90 mm

### Superior approach (Figs. 1, 3 and 5; Table 1)

**Figure 3:**
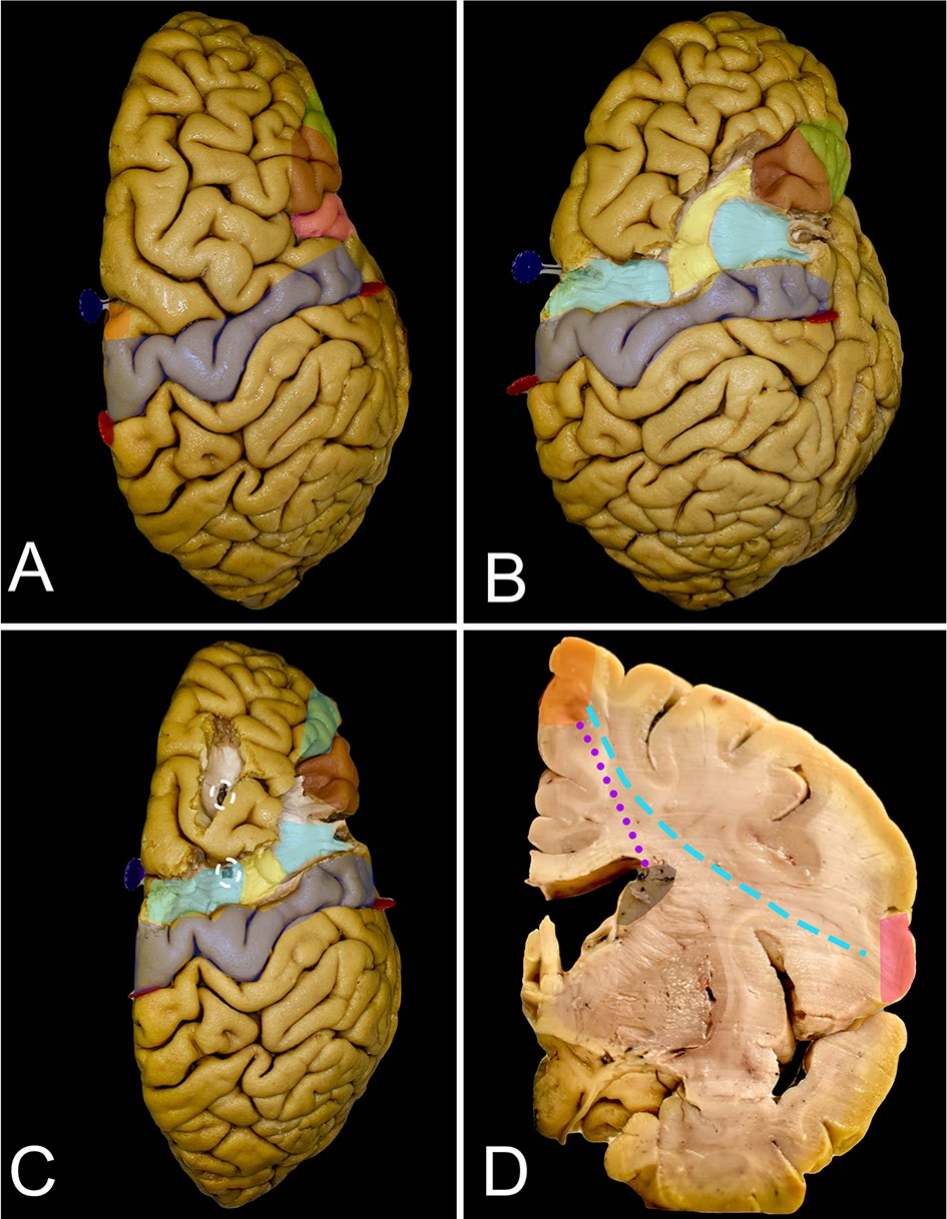
The figure presents the locations of the main white matter tracts that need to be taken into consideration when frontal lobe surgery with a superior trajectory is planned. (**A**) The key cortical regions that need to be identified are the precentral cortex (blue), SMA proper (orange), pars opercularis of the IFG (pink), pars triangularis (burgundy) and pars orbitalis (green). (**B**) Just anterior to the precentral gyrus at the level of the middle frontal gyrus, the most superficial association fibers of the AF/SLF (yellow) complex heading to the pars triangularis can be observed. Deeper in this complex are the frontal aslant tract (FAT) (light blue) fibers connecting the SMA complex on the mesial surface of the hemisphere with the pars opercularis of the IFG running almost parallel to the precentral gyrus. (**C**) The deep anatomical landmark of the resection is the frontal horn of the lateral ventricle (white circle), which can be identified within the superior frontal gyrus medial to the AF/SLF complex. (**D**) A coronal slice of the hemisphere anterior to the precentral gyrus. The caudate nucleus (black) is identified just lateral to the frontal horn. In this coronal slice, two main connections of the SMA proper in terms of language and motor function can be identified within the frontal lobe. The FAT (light blue dashed line) runs laterally to the pars opercularis, and the frontostriatal tract (FST) (purple dotted line) runs medially to the caudate nucleus.

The posterior anatomical border of the frontal lobe was defined by the central sulcus, whose main axis was projected to be approximately 70 degrees from the interhemispheric fissure (IHF) (Fig. 1D). This point can be identified approximately 5 cm posterior to the coronal suture along the interparietal suture. The width of the precentral gyrus at the level of the superor Rolandic point (SRP) was approximately 20 mm, which is two times wider than that at the base of the lobe at the inferior Rolandic point (IRP). The posterior part of the superior frontal gyrus is related to the presence of the SMA proper, which is approximately 20 mm and is anteriorly marked by the line perpendicular to the AC-PC line (anterior commissure – posterior commissure line) at the level of the AC. Aiming for the frontal lobectomy anterior to the SMA proper, the trajectory of the resection on the mesial surface must be at approximately 50 degrees to the superior margin of the hemisphere, and the genu of the corpus callosum can be reached at almost 90 mm. Following this trajectory, one would end the resection approximately 15 mm posterior to the most anterior part of the base of the frontal lobe. Changing the trajectory at that level of the genu of the corpus callosum perpendicular to the AC-PC line would extend resection for an additional 15 mm posteriorly, which would be almost half of the length of the base of the frontal lobe (Fig. 5A). On the lateral surface of the hemisphere, to achieve resection anterior to the precentral gyrus, one has to aim at an approximately 70-degree angle to the IHF, and the inferior frontal point was reached at approximately 75 mm. When assessing the bony anatomy, the end of resection from the lateral perspective is reached just posterior to the coronal suture and above the squamosal suture where the pars opercularis of the IFG is identified (Fig. 1C). At the level of the middle frontal gyrus, above the pars opercularis, the arcuate fasciculus/superior longitudinal fasciculus (AF/SLF) complex is identified at the subcortical level and runs in the caudal-rostral direction (Fig. 3B). This marks the lateral border of the resection for lesions located within the superior frontal gyrus. Posterior to the line demarcating the SMA proper from the pre-SMA, the frontal aslant tract (FAT), which connects the SMA proper with the pars opercularis, is identifiable and runs deeper into the AF/SLF complex in the dorsal–ventral direction (Fig. 1C).

**Figure 4:**
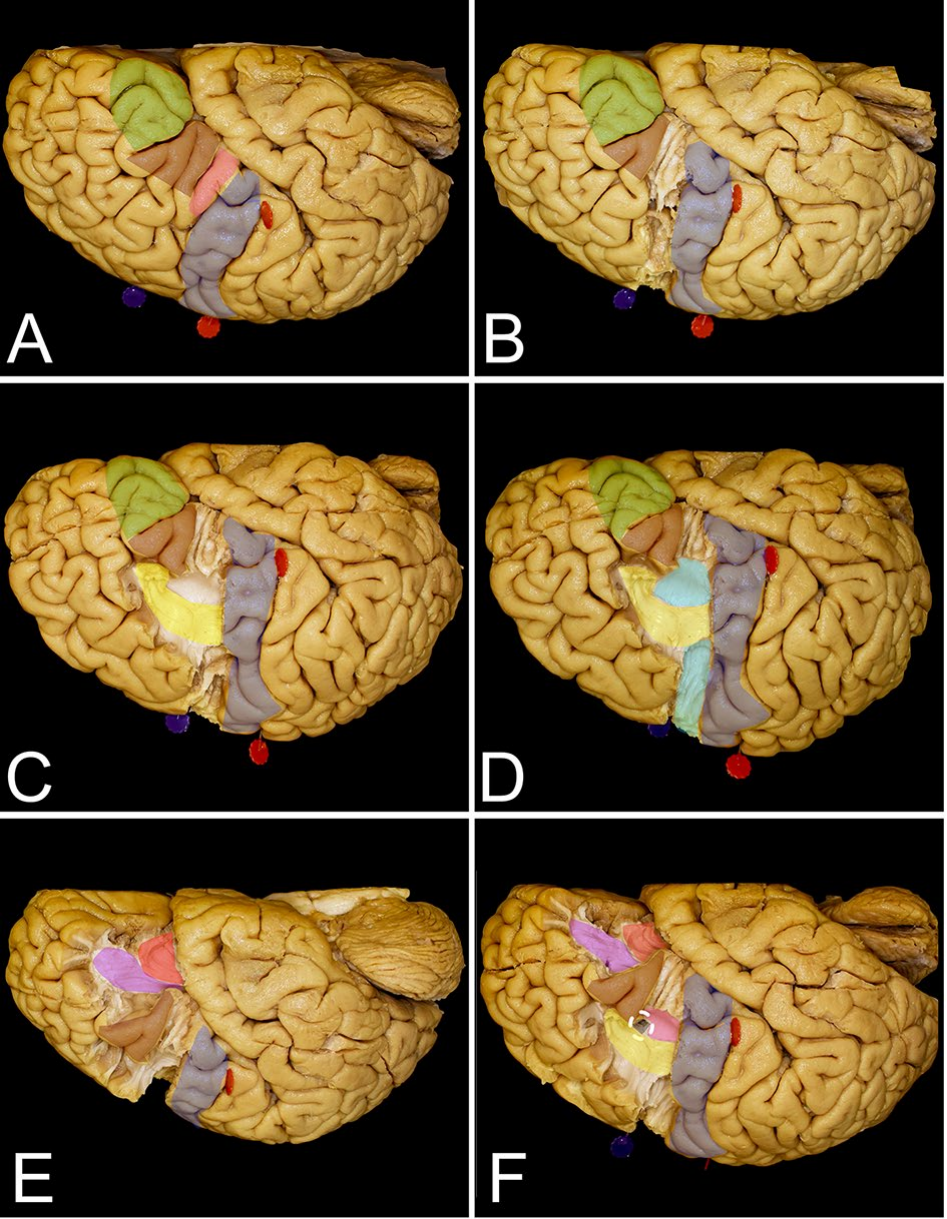
The figure presents the location of the main white matter tracts with a lateral trajectory. (**A**) The key cortical regions that need to be identified are the precentral cortex (blue), pars opercularis of the IFG (pink), pars triangularis (burgundy) and pars orbitalis (green). (**B**) The most superficial layer of the fibers, which are identified just under the cortex, is composed of the short association u-fibers that connect the neighboring gyri of the frontal lobe. Here, u-fibers within the premotor area are present after removal of the cortex just anterior to the precentral gyrus. (**C**) The most superficial bundle of long association fibers is formed by the AF/SLF complex (yellow) running around and superficial to the insular limiting sulcus. Within the frontal lobe, anterior to the precentral gyrus, this bundle can be identified at the level of the middle frontal gyrus, aiming for the pars triangularis of the IFG. The most superficial layer from the lateral perspective is formed by the second segment of the superior longitudinal fasciculus (SLF II), which connects the middle frontal gyrus to the inferior parietal lobule. The SLF III and AF terminate within the posterior parts of the inferior frontal gyrus. (**D**) Removing the AF/SLF complex in front of the precentral sulcus exposes the vertical short association white matter tract, the frontal aslant tract (FAT) (light blue), which connects the SMA complex with the pars opercularis of the IFG. The mesial part of the FAT, which connects the SMA complex with the caudate nucleus, forms the frontal striatal tract (FST)—not seen here (Fig. 3D). (**E**) The ventral long association bundle is formed by the IFOF/UF complex, terminating within the basal part of the frontal lobe. The frontal lobe is connected with the temporal pole through the UF (red) and with the parietal and occipital lobes through the IFOF (purple). This layer of white matter forms the external and extreme capsule. (**F**) From this perspective, the frontal horn of the lateral ventricle (white circle) is identified just posterior to the pars triangularis and below the AF/SLF complex.

**Figure 5:**
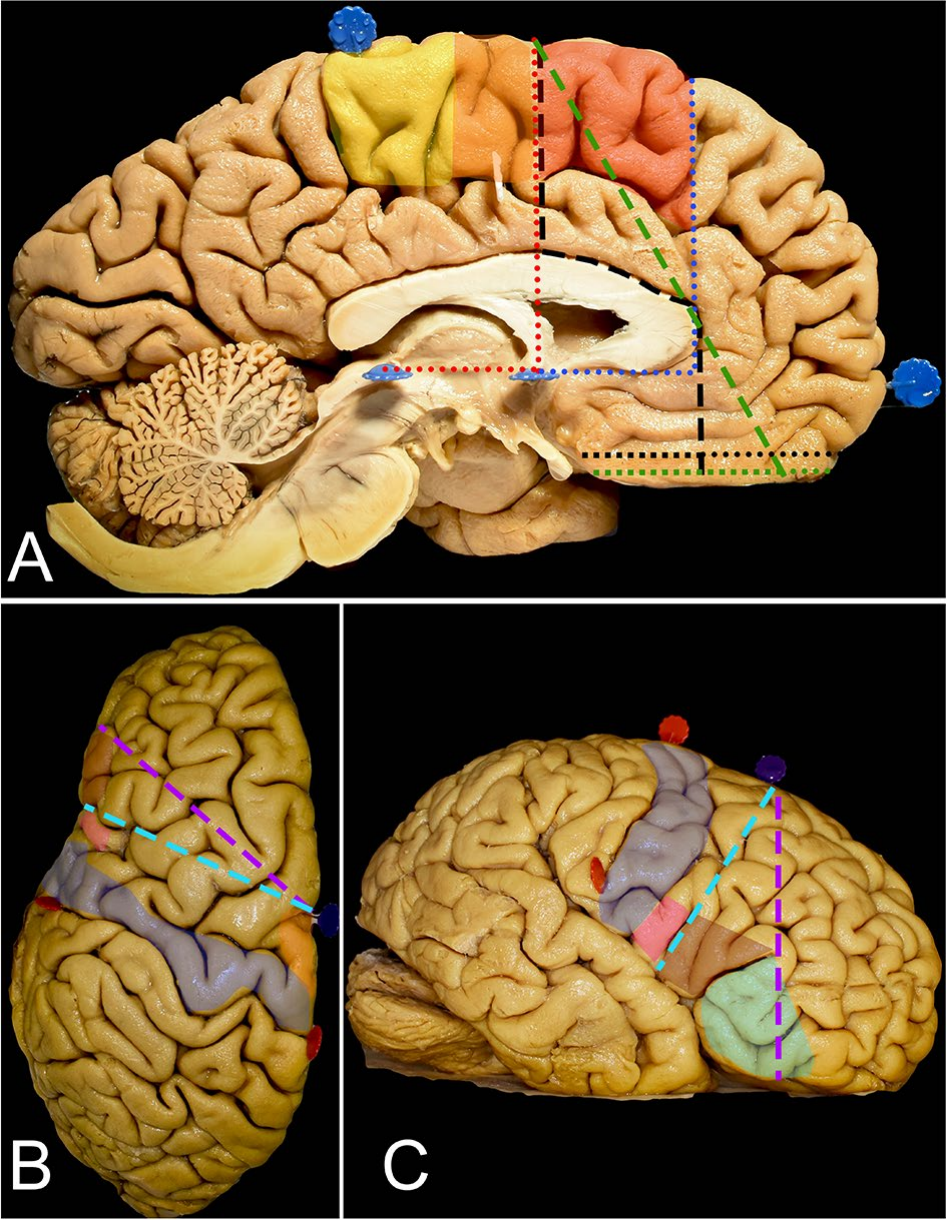
This figure presents the key aspects of frontal lobe surgery from the superior perspective. (**A**) This figure presents two techniques for resection on the mesial surface of the hemisphere. The original line (green) starts anterior to the SMA proper, and the dissection line (green dashed line) is tangential to the most rostral point of the genu of the corpus callosum. Within the base of the frontal lobe, the resection line ends within the anterior half of the gyrus rectus, leaving very short (green dotted line) segments of the gyrus rectus anteriorly, while the majority of the gyrus is left posteriorly (green dashed line); this region can be resected in the second stage after the lateral surface is resected. The modified technique (black) extends the resection up to the gyrus cinguli, going perpendicular to the brain surface from the point where the SMA proper ends to reach the corpus callosum (black dashed line). Then, the resection line moves forward along the corpus callosum up to the genu and turns again to be perpendicular to the base of the hemisphere. The resection line ends approximately at the middle of the gyrus rectus, leaving almost the same amount of the gyrus rectus anteriorly (black dotted) and posteriorly (black dashed line). The SMA proper (orange) neighbors posteriorly with the paracentral lobule (yellow) and with the preSMA (red) anteriorly. (**B**) There are also two trajectories for resection of the lateral part of the hemisphere. Without brain mapping, in the dominant hemisphere, the safer trajectory aims to be anterior to the pars triangularis (purple rectangle), while in the nondominant hemisphere, the resection can be extended posteriorly and aims to be anterior to the pars opercularis (light blue dashed line). (**C**) The same anterior (purple dashed line) and posterior (light blue dashed line) dissection lines can be applied for the lateral trajectory.

### Lateral approach (Figs. 1, 4 and 5; Table 2)

The horizontal ramus of the lateral sulcus corresponds to the inferior segment of the coronal suture, just above the squamosal suture. Posterior to these structures, the pars opercularis of the IFG is located anterior to the precentral gyrus (Fig. 1C). When performing frontal lobectomy from the lateral trajectory, the SMA proper/preSMA point is identified at approximately 85 mm along a line 80 degrees from the line parallel to the base of the frontal lobe and running parallel to the central sulcus and coronal suture starting at the level of the ascending ramus of the lateral sulcus. On the subcortical level, the dissection line in this direction runs tangential to the most rostral point of the AF/SLF complex and anterior to the anterior insular limiting sulcus. Anterior to the insula, the uncinate fascicle/inferior fronto-ocipital fascicle (UF/IFOF) complex was identified at a depth of approximately 25 mm from the cortical surface (Fig. 4E). Passing the UF/IFOF complex, the midline is reached in the next 25 mm along the base of the frontal lobe, passing through the olfactory groove 7 mm before midline. Above the AF/SLF complex, the posterior border of the dissection line is marked by the FAT (Fig. 4D).

### Illustrative cases

#### Case 1 (Fig. 6)

A 44-year-old right-handed man presented with an asymptomatic lesion characteristic of a LGG that was diagnosed with a full body MRI scan performed as a routine check-up on patient demand. The lesion was located within the dominant inferior frontal gyrus, being anterior to the AF/SLF complex, and the FAT while being lateral to the IFOF. In the preoperative neuropsychological assessment, the patient presented with slight deficits in working memory and attention. The patient was operated in awake conditions in the lateral position with the head rotated to the right. Intraoperative electrical stimulation of the cortex posterior to the tumor elicited negative responses during picture naming tasks. These areas based on neuronavigation were related to the cortical terminations of the AF/SLF complex. In the postoperative period, patient presented without neurological deficits, while in the neuropsychological assessment increased preoperative deficits in working memory and attention were observed with additional subtle deficits in executive functions. The neuropsychological deficits improved gradually in the long-term follow-up. The final neuropathology was WHO grade II astrocytoma.

**Figure 6:**
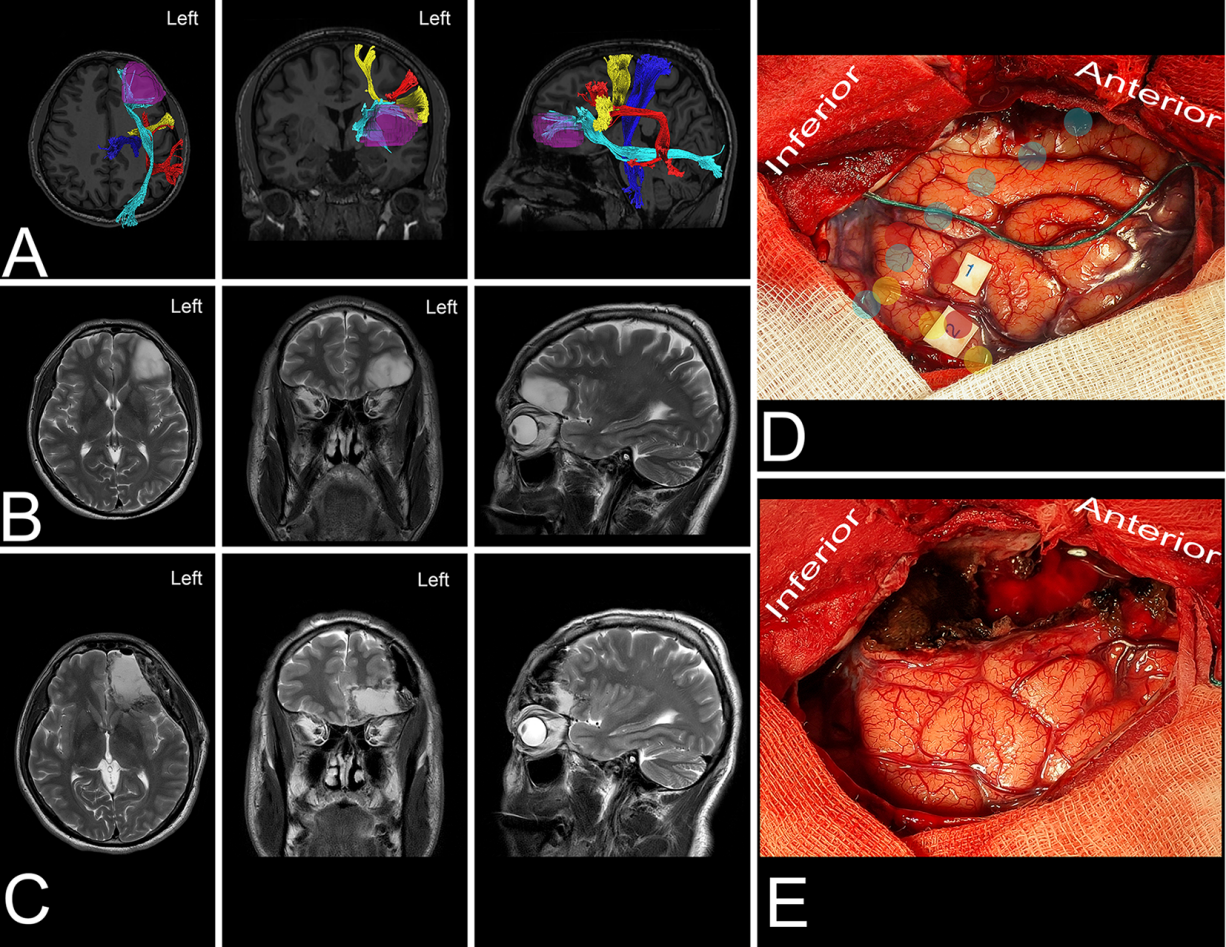
The figure presents a 44-year-old right-handed man with a LGG within the dominant frontal lobe. (**A**) Axial, coronal, and sagittal MRIs with diffusion tensor imaging tractography reconstruction of the tracts passing within the frontal lobe and related to the tumor. The tumor (purple) is anterior to the AF/SLF complex (red), FAT (yellow) and corticospinal tract (blue) and lateral to the IFOF (light blue). (**B,C**) Axial, coronal, and sagittal MRIs with preoperative and postoperative imaging of the tumor located within the inferior frontal gyrus. (**D**) Intraoperative photograph taken before tumor removal and after brain mapping. The tumor (anterior to green marker) was located anterior to the Broca’s area (tags 1 and 2). The tracts related to the resection are marked—AF/SLF complex (red dots), FAT (yellow) and IFOF (light blue). (**E**) Intraoperative photograph taken after tumor resection within functional bordersThe patient underwent surgery in the lateral position with the head rotated to the right. The posterior border of the resection on the cortical level was marked with stimulation where arrest of speech or movement (marker 2) and aphasia (marker 1) was observed. The posterior dissection plane was marked by arachnoid, while deeper on the subcortical level the stimulation was performed in the region where the AF/SLF complex was expected, and aphasia was observed. Anterior the resection was extended to the pars orbitalis, superior to the inferior frontal sulcus while at depth to the gyrus rectus and IFOF. DTI was reconstructed in DSI Studio (http://dsi-studio.labsolver.org)^37^.

#### Case 2 (Fig. 7)

A 31-year-old right-handed man presented with a history of seizures without neurological and cognitive deficits. MRI revealed a lesion characteristic of a LGG within the dominant superior frontal gyrus, being anterior to the SMA/FAT and medial to the AF/SLF complex. The patient was operated on in the semisitting position with the head in the neutral position, under awake conditions. Intraoperative electrical stimulation of the cortex posterior to the tumor elicited responses from the primary motor cortex. Stimulation of the white matter at the posterior border of resection elicited movement and naming arrest. These areas based on neuronavigation were related to the FAT. In the postoperative period, the patient presented with symptoms of transcortical motor aphasia, ideomotor apraxia and subtle deficits in attention which improved in the long-term follow-up. The final neuropathology was WHO grade II astrocytoma.

**Figure 7:**
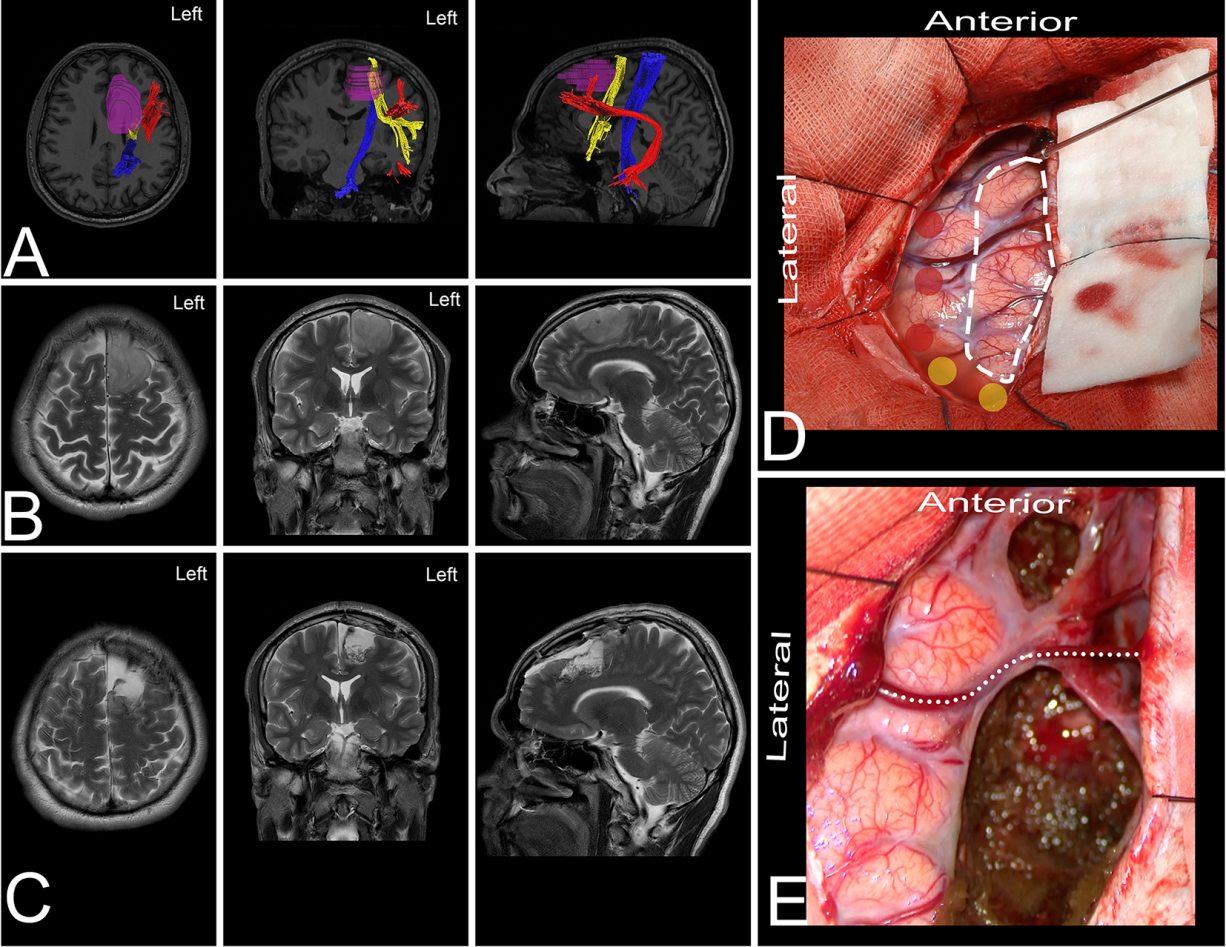
The figure presents a 31-year-old right-handed man with a LGG within the dominant frontal lobe. (**A**) Axial, coronal, and sagittal MRIs with diffusion tensor imaging tractography reconstruction of the tracts passing within the frontal lobe and related to the tumor. The tumor (purple) is anterior to the FAT (yellow) and corticospinal tract (blue) and medial to the AF/SLF complex (red). (**B,C**) Axial, coronal, and sagittal MRIs with preoperative and postoperative imaging. (**D**) Intraoperative photograph taken before tumor removal and after brain mapping. The tumor (white rectangle) was located anterior to the primary motor cortex and SMA. The tracts related to the resection are marked—AF/SLF complex (red dots) and FAT (yellow). (**E**) Intraoperative photograph taken after tumor resection within functional borders with two corticotomies to preserve the draining vein (white dots). The patient was operated on in the semissitting position with the head in the neutral position. The extent of resection on the lateral, mesial and anterior border was marked by the arachnoid of the surrounding sulci while the posterior and inferior were marked by the result of the subcortical stimulation where the arrest of speech and movement was observed. DTI was reconstructed in DSI Studio (http://dsi-studio.labsolver.org)^37^.

#### Case 3 (Fig. 8)

A 34-year-old right-handed man presented with a history of seizures without neurological deficits. MRI revealed a lesion characteristic of a LGG within the nondominant frontal lobe, anterior to the coronal suture and anterior to the FAT and corticospinal tract. In the neuropsychological assessment, the patient presented impaired executive functions attention, memory, and visuospatial functions. The patient underwent surgery under general anesthesia in the semisitting position with the head in the neutral position. Anatomical resection of the frontal lobe starting at the level of the coronal suture aiming for the ridge of the lesser sphenoid wing was performed. Resection based on neuronavigation was performed anterior to the cortico-spinal tract and the FAT. In the postoperative period, the patient was neurologically intact while in the neuropsychological assessment increased deficit in executive functions, especially in terms of drive, emotional and behavioral self-regulation, reasoning, planning and inhibition were observed. Additionally, compared to the preoperative state moderate deficits in memory, attention and visuospatial function occurred. All observed deficits gradually improved. The final neuropathology was WHO grade II/III astrocytoma.

**Figure 8:**
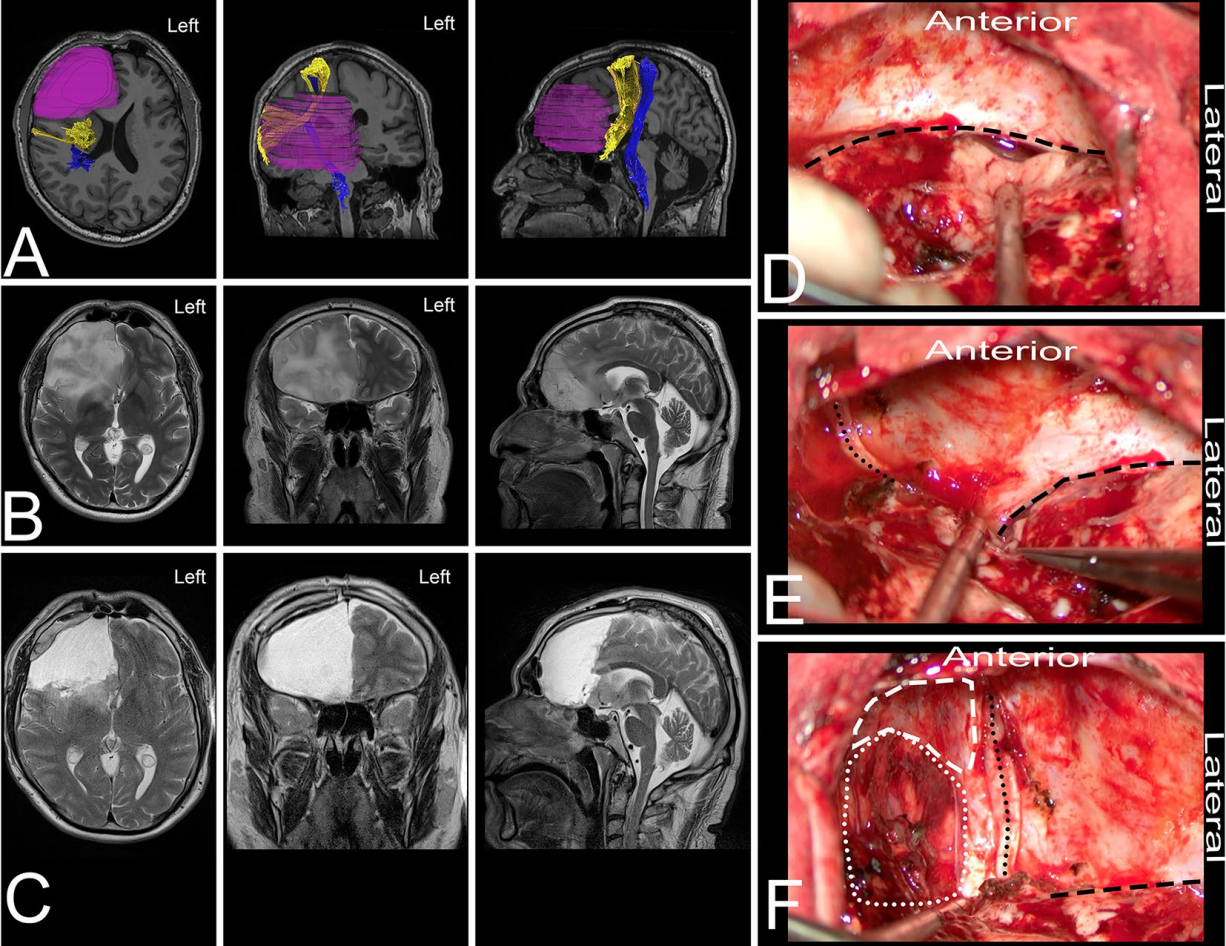
The figure presents a 34-year-old right-handed man with a LGG within the nondominant frontal lobe. (**A**) Axial, coronal, and sagittal MRIs with diffusion tensor imaging tractography reconstruction of the tracts passing within the frontal lobe and related to the tumor. The tumor (purple) is anterior to the FAT (yellow) and corticospinal tract (blue). (**B,C**) Axial, coronal, and sagittal MRIs with preoperative and postoperative imaging. (**D–F**) Intraoperative photographs taken after frontal lobectomy from lateral to medial. The resection started at the level of the coronal suture and aimed to reach the free ridge of the lesser sphenoid wing (black rectangle) with preservation of the olfactory tract within the arachnoid membrane (black dots). Medially, the falx cerebri (white rectangle) and contralateral frontal lobe (white dots) were identified. The patient was operated on in the semisitting position with the head in the neutral position. DTI was reconstructed in DSI Studio (http://dsi-studio.labsolver.org)^37^.

## Discussion

### The primary motor cortex

Permanent motor deficits related to frontal lobe surgery are related to injury to the cortical structures within the precentral gyrus–primary motor cortex. The traditional posterior bony landmark to avoid permanent motor deficits during frontal lobectomy is the coronal suture, which is localized approximately 13 cm from the nasion^13^. The coronal suture is located approximately 3.8 inferiorly and 5.3 cm superiorly anterior to the central sulcus and 2.3 inferiorly and 3.6 cm superiorly anterior to the precentral sulcus^14^. The main axis of the central sulcus is approximately 70 degrees anterior to the IHF, making the IRP closer than the SRP to the coronal suture, which is almost in the coronal plane. From a practical point of view during frontal lobectomy to avoid ventricular system and motor deficits the trajectory of the resection should be aiming from the coronal suture to the ridge of the lesser sphenoid wing which can easily be guided by the neuronavigation system (Case 3). Intraoperative stimulation of the precentral cortex and corticospinal tract subcortically, both during awake and asleep conditions, the results in positive responses, and muscle contraction within the face or extremities can be observed. In terms of awake craniotomy, it is important to expose the central sulcus to set the lowest stimulation thresholds and minimize the risk of intraoperative seizures^15,16^.

### Supplementary motor complex (SMC)

The supplementary motor complex (SMC) is identified on the mesial and lateral surface of the hemisphere anterior to the primary motor cortex. The SMC is composed of the SMA proper posteriorly, preSMA anteriorly and supplementary eye fields (SEFs) located in-between^17^. In addition, there is no clear anatomical definition of the premotor cortex, and the SMA complex is defined as a region on the mesial surface of the hemisphere within the superior frontal gyrus just anterior to the paracentral lobule. The border between the SMA proper and pre-SMA complex is established by an imaginary line passing through the anterior commissure perpendicular to the line connecting the anterior and posterior commissure. The anterior margin of the pre-SMA is even less defined, but some define it as a line parallel to the imaginary line at the most rostral point of the genu of the corpus callosum^18^. Based on our study, the anterior border of the SMA proper is located 17.3 mm anterior to the central sulcus along a line almost perpendicular (mean 87.5°) to the superior margin of the hemisphere. Intraoperative stimulation within the SMA complex results in negative responses presenting as a weakness, delay of movement initiation, slowness of movement, difficulties performing dual tasks or coordination disturbance^19^. On the subcortical level, the main output of the SMC in terms of motor and language function is the frontal aslant tract/frontal striatal tract (FAT/FST) complex^20^. There is ongoing debate about the usefulness of intraoperative mapping for the FAT/FST complex during awake conditions and the functional benefits of preservation in terms of neurooncological outcome due to mostly temporary deficits related to its resection^21,22^. The FAT connects the SMA complex and pars opercularis of the IFG, while the FST connects the SMA complex with the caudate nucleus^21,23^. This network was confirmed with in-vivo studies based on cortico-cortical evoked potentials (CCEPs)^24^. This technique also allows for intraoperative mapping and monitoring of language function under general anesthesia^25^. Intraoperative stimulation of FAT results in stuttering, speech arrest or problems with verb generation, while FST stimulation results in negative motor responses^21^. In the presented illustrative Case 2 intraoperative stimulation of the white matter at the posterior border of the resection resulted in arrest of speech and movement. Deficit was temporary and resolved in long term follow-up. The recovery mechanism in these patients is explained by crossed transcallosal fibers and recruitment of the contralateral, nondominant hemisphere^11,12,26^. This fiber system runs parallel to the precentral sulcus aiming for the pars opercularis of the IFG, mesial to the AF/SLF complex at the level of the middle frontal gyrus.

### The pars opercularis

The pars opercularis is the most posterior compartment within the IFG, anterior to the precentral sulcus, behind the coronal suture and below the superior temporal line, and this structure measures approximately 7.5 mm according to present data^27^. Pars opercularis forms part of the ventrolateral premotor cortex, which should be preserved, especially on the dominant hemisphere, and limited brain plasticity within this region makes it often nonresectable^28^. Intraoperative stimulation of the dominant ventrolateral premotor cortex results in anarthria, while stimulation of the dorsolateral part results in anomia^29^. In terms of movement, intraoperative stimulation of the premotor cortex results in interruption of movement execution and alters patients’ awareness of movement^30^. Within the premotor cortex where the middle frontal gyrus connects with the precentral sulcus lies the frontal eye field. The superior and inferior frontal points that mark the posterior ends of the middle frontal gyrus are located 27 and 51 mm from the IHF, respectively. Intraoperative stimulation of the frontal eye field results in saccadic eye movement, while cortical stimulation anterior to it may result in writing disabilities such as writing arrest or letter omissions^31^. This effect is related to Exner’s area within the dominant hemisphere, which seems to be involved in the cognitive aspects of writing and reading^32^. The pars opercularis and anteriorly located pars triangularis constitute the so-called Broca’s speech area, which stimulates arrest of speech and anomia and is approximately 28.3 mm in length in the widest dimension. The anterior 20.2 mm corresponds to the pars triangularis. Within this region, the frontal terminations of the AF/SLF complex can be identified^20^. The AF/SLF complex represents the dorsal language pathway, which is the main tract related to language; when stimulated intraoperatively, it may result in speech production disorders of anomia and phonemic paraphasias and articulation disorders. The ventral language pathway is represented by the UF/IFOF complex, which is identified at the inferior-anterior border of the resection, mainly within the pars orbitalis of the frontal lobe^33^. Intraoperative brain stimulation during naming tasks can identify the UF/IFOF complex, as it produces semantic paraphasia in the dominant hemisphere and nonverbal semantic disturbances in the nondominant hemisphere. Resection of tumors located within the IFG is usually safe when performed anterior to the pars opercularis as presented in Case 1.

### Deep grey mater structures

The single constant anatomical landmark within the frontal lobe is the frontal horn of the lateral ventricle. The level of the inferior frontal sulcus laterally corresponds to the superior margin of the corpus callosum medially, and the line connecting both points in the coronal section is tangent to the roof of the lateral ventricle. Below this line, the head of the caudate and lentiform nucleus can be identified. Intraoperative stimulation of the lentiform nucleus on both sides generates articulation disorders, while stimulation of the head of the dominant caudate nucleus results in perseveration during naming tasks^34^. Both structures can be encountered with the posterior trajectory of the resection above and at the level of the insula. From a surgical perspective, it is worth noting that the tip of the ascending ramus of the lateral sulcus points into the anterior insular point, which is identified anterior to the basal ganglia. From a superior perspective, when the frontal horn of the lateral ventricle is opened, extension of the resection to the base of the frontal lobe through the most anterior-inferior point of the frontal horn guides the resection approximately to the level of the vertical limb of the orbital sulcus^35^. Despite the abovementioned deficits, resections of the so-called prefrontal cortical and subcortical regions of the frontal lobe are related to executive function and behavioral changes^34^.

The present results allowed systematization of the general superficial and white matter organization of the frontal lobe combining neuroanatomical basic science with neurosurgical perspective. Better understanding of the different anatomical layers of the frontal region, including craniometric points are crucial when planning surgery and strategies of intraoperative brain mapping what is essential in improving surgical and functional outcome. To the best of our knowledge, there is no such studies which combine all these aspects of the frontal lobe surgery with morphometrics measurements and anatomical dissections.

### Limitations of the study

The physical parameters of the cadaveric brain tissue do not represent the intraoperative brain structure. The anatomical relationship between cortical and white matter structures in patients with tumors is disturbed by the tumor mass or edema. A better surgical perspective of the operation field would be obtained with specimens containing preserved arterial and venous system vessels as well as preserved cranial structures. Additionally, when resections are based on function it cannot be related to the purely anatomical study.

## Methods

Ten adult cerebral hemispheres with no intracranial pathology or description of neurological disease in their medical history were fixed with 4% formalin for at least 4 weeks. The dry brain, with arachnoid and vessels removed, was placed inside the freezer with at a temperature of –15 degrees Celsius for 2 weeks. For thawing and subsequent preservation, a 4% formalin solution at room temperature was used. The details of different modifications of the Klingler technique were described previously^36^. For dissections, the brain was placed in a position simulating the intraoperative scenario. For lesions in the SMA region and superior trajectory, the long axis of the brain was perpendicular to the floor, while for lesions in the frontal operculum and lateral trajectory, it was parallel to the floor (Fig. 1). The assessment of cortical anatomy was performed with the naked eye, while white matter dissection was performed in a stepwise manner with microscopic magnification (Fig. 2). The measurements included the distance between the constant anatomical cortical landmarks within the frontal lobe, white matter tracts and ventricular system. The localization of the tracts in relation to the main cortical landmarks was also taken into consideration. Measurements were made with an electric digital caliper, protractor and measuring tape. A digital camera (NikonD7200 with a Nikon DX 35 mm lens 1:1.8G) was used for image documentation. Color markers seen in the figures correspond to constant anatomical points and were placed for better anatomical orientation when the position of the brain or observation perspective was changed. Illustrative clinical cases present three different patients with LGG located within the frontal lobe (inferior frontal gyrus, superior frontal gyrus, whole frontal lobe) which may benefit from surgery with direct brain stimulation in terms of the extent of resection. The neuropsychological assessment of the patients was performed two days before the procedure, within a week and after a month. The diffusion images presented in the study were acquired on a GE SIGNA Architect scanner using a diffusion sequence. TE = 77.2 ms, and TR = 9541 ms. A DTI diffusion scheme was used, and a total of 63 diffusion sampling directions were acquired. The b-value was 1000.57 s/mm^2^. The in-plane resolution was 1.0156 mm. The slice thickness was 2 mm.

The study was approved and the informed consent was waived by the Bioethics Committee of Medical University of Warsaw; the approval number is AKBE/126/2019. Informed consent for clinical data was obtained from all presented subjects. All procedures performed in studies involving human participants were in accordance with the ethical standards of the institutional and/or national research committee and with the 1964 Helsinki declaration and its later amendments or comparable ethical standards.

### Ethical approval

The study was approved by the Bioethics Committee of Medical University of Warsaw; the approval number is AKBE/126/2019. All procedures performed in studies involving human participants were in accordance with the ethical standards of the institutional and/or national research committee and with the 1964 Helsinki declaration and its later amendments or comparable ethical standards.

## Data Availability

The full data is available in the manuscript.
